# A 20-year prospective study of mortality and causes of death among hospitalized opioid addicts in Oslo

**DOI:** 10.1186/1471-244X-8-8

**Published:** 2008-02-13

**Authors:** Mari A Bjornaas, Anette S Bekken, Aasa Ojlert, Tor Haldorsen, Dag Jacobsen, Morten Rostrup, Oivind Ekeberg

**Affiliations:** 1 Department of Acute Medicine, Ullevaal University Hospital, N-0407 Oslo, Norway; 2 Department of Behavioural Sciences in Medicine, Institute of Basic Medical Sciences, University of Oslo, N-0317 Oslo, Norway; 3 Department of Biostatistics, Institute of Basic Medical Sciences, University of Oslo, N-0317 Oslo, Norway

## Abstract

**Background:**

To study mortality rate and causes of death among all hospitalized opioid addicts treated for self-poisoning or admitted for voluntary detoxification in Oslo between 1980 and 1981, and to compare their mortality to that of the general population.

**Methods:**

A prospective cohort study was conducted on 185 opioid addicts from all medical departments in Oslo who were treated for either self-poisoning (*n *= 93, 1980), voluntary detoxification (*n *= 75, 1980/1981) or both (*n *= 17). Their median age was 24 years; with a range from 16 to 41, and 53% were males. All deaths that had occurred by the end of 2000 were identified from the Central Population Register. Causes of death were obtained from Statistics Norway. Standardized mortality ratios (SMRs) were computed for mortality, in general, and in particular, for different causes of death.

**Results:**

During a period of 20 years, 70 opioid addicts died (37.8%), with a standardized mortality ratio (SMR) equal to 23.6 (95% CI, 18.7–29.9). The SMR remained high during the whole period, ranging from 32.4 in the first five-year period, to 13.4 in the last five-year period. There were no significant differences in SMR between self-poisonings and those admitted for voluntarily detoxification. The registered causes of death were accidents (11.4%), suicide (7.1%), cancer (4.3%), cardiovascular disease (2.9%), other violent deaths (2.9%), other diseases (71.4%). Among the 50 deaths classified as other diseases, the category "drug dependence" was listed in the vast majority of cases (37 deaths, 52.9% of the total). SMRs increased significantly for all causes of death, with the other diseases group having the highest SMR; 65.8 (95% CI, 49.9–86.9). The SMR was 5.4 (95% CI, 1.3–21.5) for cardiovascular diseases, and 4.3 (95% CI, 1.4–13.5) for cancer. The SMR was 13.2 (95% CI, 6.6–26.4) for accidents, 10.7 (95% CI, 4.5–25.8) for suicides, and 28.6 (95% CI, 7.1–114.4) for other violent deaths.

**Conclusion:**

The risk of death among opioid addicts was significantly higher for all causes of death compared with the general population, implying a poor prognosis over a 20-year period for this young patient group.

## Background

Mortality rates among opioid addicts are higher than for the general population, although there are differences between countries and regions [1]. Standardized mortality ratios (SMRs) vary between 15 and 28 in different studies [2-4]. In spite of the increased mortality, and hence the need for intervention, Cook et al. found that 67% of patients who attended an emergency department because of acute opioid overdose were not referred to any therapeutic programme for drug addiction [5]. During the last few decades, studies have focused on abstinence versus continued drug use following participation in various treatment programmes, as an outcome measure [6], even though there is still some doubt about whether such programmes have a long-term effect on mortality. In a 15-year follow-up study, Sorensen et al. found that people who had achieved stable abstinence from injectable narcotics use were at lower risk of premature death when compared with those who continued using drugs [7]. However, even in the presumed abstinence group, there was a sevenfold increase in SMR. This raises the question whether referral to a detoxification programme has a satisfactory effect on long-term mortality.

Although the majority of deaths among opioid addicts are from accidental poisonings [8], deaths from other unnatural causes, such as suicide, have also increased [9,10]. The co-morbidity between drug dependence and suicide has been described both as an association between drug dependence and mental illness [11], and between known risk factors for both drug dependence and suicide, such as gender, living alone and unemployment [12]. In an empirical review, Wilcox et al. estimated the SMR for suicide associated with opioid dependence, but did not estimate SMRs for other diseases [10]. In another empirical review, Harris and Barraclough calculated SMRs for both natural and unnatural causes of death associated with opioid dependence [13].

Review studies, which have proven useful in pointing out the increased mortality in this patient group, have used expected number of deaths based on WHO data, rather than patient data. The analyses in Harris and Barraclough's review were based on 10 papers with a follow-up period ranging from 1 to 28 years, and the SMRs for natural causes of death were based on only one study. Longer prospective follow-up studies of opioid addicts show how the overall mortality rate changes over time [14], it being expected that cause-specific mortality would also differ between long-term and short-term follow-up studies. Although cause-specific mortality rates were measured in one four-year prospective cohort study, no SMRs were obtained [15]. Prospective cohort studies in the field are relatively uncommon [15], and even rarer is the opportunity to compare the cohort with a well-defined background population that includes a whole city. However, long-term prospective studies of excess mortality among opioid addicts, in particular, would be useful for obtaining more specific information about opioid addictions as a subgroup of substance use disorders.

During recent decades, the AIDS epidemic has had a major effect on this group of patients [16]. Although mortality among opioid addicts increased from 1980 to 1988 [17], this increase can only be partly explained by the emergence of AIDS [18]. Cause-specific SMRs during recent decades would be helpful in studying mortality in this group of patients.

The aims of this study were to study the mortality rate and causes of death among opioid addicts who had been treated for self-poisoning or admitted to voluntary detoxification in 1980 and 1981 during a 20-year follow-up investigation. The study compared this cohort with the general population. The study's design allowed an investigation of whether participation in a detoxification programme can be protective.

## Methods

### Participants

The study included all opioid addicts hospitalized due to self-poisoning in Oslo in 1980, as well as all opioid addicts admitted for voluntary detoxification in 1980 and 1981 (Table 1). All patients who were discharged following treatment for self-poisoning and classified as opioid abusers in 1980 in Oslo (454,000 inhabitants) medical departments, were included. This subgroup was obtained from a larger prospective cohort study that included all patients treated for self-poisoning in Oslo medical departments in 1980 [19,20]. Patients leaving directly from the emergency room after treatment were also included in the study. The first, or the most serious, admission (i.e., according to the toxic agent used and the amount taken) was chosen as the index episode.

**Table 1:** Opioid addicts treated at medical departments in Oslo in 1980/1981

	**Self-poisonings *n *= 93**	**Voluntary detoxification *n *= 92**	**Total *n *= 185**
**Females**	32	54	86 (46.5%)
**Males**	61	38	99 (53.5%)
**Median age**	24	23	24
**Average number of admittances**	1.4	1.4	1.4

All opioid addicts admitted to Ullevaal University Hospital for voluntary detoxification during 1980 and 1981 were included in the study. During the late 1970s, the number of opioid addicts increased rapidly in Oslo, particularly among young people. The treatment facilities were rather poor. Therefore, from 1980, the Department of Acute Medicine (previously Medical Department 7) accepted the responsibility for treating up to three opioid addicts simultaneously. These people were in need of detoxification, either before further treatment in drug addiction units, or due to their poor overall health, Ullevaal University Hospital serving this function for the whole city [21]. Before admittance, volunteer patients were interviewed to obtain their agreement regarding the ward's rules, and they were informed that no opioids or benzodiazepines would be prescribed. They were offered neuroleptics and medication, such as alimemazin, which served as hypnotics. Their health contact person was usually present on admission. They had daily meetings with a social worker or a psychiatrist for support and planning further treatment. The staff at the units received regular supervision. About 75% of those patients who were detoxified were transferred for further planned treatment programmes. About 50% of those in a bad physical condition, left the unit before they were advised to do so. The average length of stay for the total group was 3.5 days. Although patients who had experienced an opioid drug overdose were offered interviews with a social worker or a psychiatrist, most of them were not interested in further treatment and left the hospital when they were able to do so.

The present cohort contained 185 opioid addicts treated for either self-poisoning (*n *= 93), voluntary detoxification (*n *= 75), or both (*n *= 17). Those who were admitted initially for self-poisoning and later for detoxification were classified in further analyses as voluntarily detoxifications, to indicate their willingness to undergo further treatment and rehabilitation. Some patients were hospitalized several times (Table 2). Since information about the status of the patients at discharge was only available for groups and not individuals, the statistical significance of this variable was not investigated. There were 99 (53.5%) males and 86 (46.5%) females. Their median age was 24 years (range 16–41), 72% of patients being in the 20–29 year age group (*n *= 133).

**Table 2:** Number of self-poisonings and voluntary detoxifications among the cohort of opioid addicts during 1980/1981

**Group**	***n***	**%**
**1 self-poisoning**	71	38.4
**> 1 self-poisoning**	22	11.9
**self-poisoning *and *voluntary detoxification**	17	9.2
**1 voluntary detoxification**	58	31.4
**> 1 voluntary detoxification**	17	9.2

**Total**	185	100.1

### Design

Classification of abuse was based on patient interviews and records. The basic criteria were information about daily, or regular, use of the respective compounds, including withdrawal symptoms if the compounds were not administered.

The causes of death were classified according to the death certificates provided by Statistics Norway and grouped according to the appropriate standards. Thus, the same classification was used for both the study population and the general population. Death certificates were based on forensic autopsy records for all cases that did not involve natural causes [22]. Appropriate categories from the International Classification of Diseases (ICD) were used for deaths occurring before 1987 (ICD-8), for deaths from 1987 to 1996 (ICD-9), and for deaths from 1996 to 2000 (ICD-10). All deaths were categorized according to their major cause: cardiovascular disease, cancer, other diseases, suicide, accidents, and other violent deaths. The category, other diseases, consisted of all diseases except for cardiovascular diseases and cancer. Other violent deaths consisted of all external causes of injury, except for suicides and accidents. There was no specific cause of death for four patients, three deaths being recorded as 799 unknown (ICD-9), and one being recorded as R999 unknown (ICD-10). These cases were all classified as other diseases.

Mortality in the cohort was compared with that of the general population of Norway. Reference rates were computed in terms of five-year calendar periods and five-year age groups (15–19, 20–24, etc.) for each gender. Patients were followed to emigration, death or the end of 2000, whichever occurred first. Dates of emigration and death were obtained from the Central Population Registry. Person-time under observation, categorized by five-year calendar periods and five-year age groups (15–19, 20–24, etc.), was obtained for each gender. Mortality rates in the general population for the same cross-classification served as reference rates. The expected number of deaths was computed by multiplying the observed person-time in the cohort by the reference rates for each cell in the cross-classification, and appropriately adding the results. SMRs were computed by taking the ratio of observed to expected deaths (i.e., the ratio of the number of deaths observed in the study group to the number of deaths that would be expected if the study population had the same specific rates as the standard population).

### Data analyses

The 95% confidence interval (95% CI) was computed for each SMR, based on the assumption that observed deaths follow a Poisson distribution. Stata release 8.0 programmes were used for survival analysis (Stata Corporation). For other data analyses, SPSS version 14.0 was used (SPSS Inc.). Comparisons between groups were performed using chi-squared tests, *P *< 0.05 being considered statistically significant.

### Ethics

Permission for both the original cohort study and the follow-up study was obtained from the National Data Inspectorate and the Regional Ethics Committee, both of which agreeing that informed consent was not necessary. Asking the patients' permission in advance to check whether they had committed suicide or had died from other causes during a 20-year follow-up period was considered unethical. All data were stored anonymously by substituting a number code for each patient's name, and placing the data in the locked office of the head physician.

## Results

### Mortality rates

During the 20-year follow-up period, 70 of the patients died (37.8%), including 23 females (31.4%) and 47 males (48.5%). Five patients (2.7%) had emigrated, and 110 patients (59.5%) were still alive. The overall SMR was 23.6 (95% CI, 18.7–29.6). The SMR was 23.4 (95% CI, 17.6–31.1) for males, and 24.2 (95% CI, 16.1–36.4) for females, this difference between males and females being non-significant. SMR was increased in each successive five-year period following the initial self-poisoning (Table 3), the highest SMR occurring in the first five years, 32.4 (95% CI, 21.3–49.1). The median age at death was 34 years, with a range from 20 to 58.

**Table 3:** Deaths and standardized mortality ratios (SMRs) for opioid addicts for each five-year period

	**Self-poisonings**		**Voluntary detoxification**		**Total**	
	**Deaths**	**SMR (95% CI)**	**Deaths**	**SMR (95% CI)**	**Deaths**	**SMR (95% CI)**

**Period 1**	12	28.8 (16.3–50.7)	10	38.0 (20.5–70.7)	22	32.4 (21.3–49.1)
**Period 2**	11	28.2 (15.6–51.0)	9	31.1 (16.2–59.7)	20	29.5 (19.0–45.7)
**Period 3**	11	27.5 (15.5–49.7)	5	16.0 (6.7–38.4)	16	22.5 (13.8–36.7)
**Period 4**	6	12.1 (5.4–26.9)	6	15.1 (7.0–33.7)	12	13.4 (7.6–23.7)

**Total**	40	23.5 (17.2–32.0)	30	23.8 (16.6–40.0)	70	23.6 (18.7–29.9)

### Differences between patients treated for self-poisoning and those treated voluntarily for detoxification

Forty patients (43.0%) died among those solely discharged after self-poisonings, and 30 patients (48.4%) died among those treated voluntarily for detoxification (Table 3). The SMR was 23.5 (95% CI, 17.2–32.0) for those discharged after an episode of self-poisoning, whereas the SMR was 23.8 (95% CI, 16.6–34.0) for those who were admitted voluntarily for detoxification. The difference in SMR between groups was not statistically significant, nor were there any significant gender differences. For both groups, mortality was highest during the first five years after admittance, and remained high during the whole period thereafter.

### Causes of death

The main causes of death were categorized as accidents (*n *= 8, 11.4%), suicide (*n *= 5, 7.1%), cancer (*n *= 3, 4.3%), other violent deaths (*n = *2, 2.9%), cardiovascular disease (*n *= 2, 2.9%), and other diseases (*n *= 50, 71.4%). The other diseases category compromised the vast majority of deaths. In 37 cases (52.9% of the total), the cause of death was classified in ICD-8 and ICD-9 as category 304, Drug dependence (Table 4), and listed according to ICD-9 as a natural cause of death.

**Table 4:** Deaths during follow-up that were classified as other diseases. The deaths are further specified according to the ICD systems.

**Code**	**Title**	***n***	**%**
304	Drug dependence	37	74.0
305	Non-dependent abuse of drugs	2	4.0
279	Disorders involving the immune mechanism	3	6.0
B20.1	HIV	1	2.0
F11.0	Psychiatric diseases and behavioural diseases caused by opiate dependence	1	2.0
F12.0	Mental and behavioural disorders due to use of cannabinoids	1	2.0
F19.0	Psychiatric diseases and behavioural diseases caused by multiple drug dependence	1	2.0
799	Unknown	3	6.0
R999	Unknown	1	2.0

**Total**		**50**	**100**

SMRs were significantly increased for all causes of death (Table 5). The other diseases category had the highest SMR, 65.8 (95% CI, 49.9–86.9). Accidental poisonings, that is overdoses, were included in the accidents category, with an SMR equal to 13.2 (95% CI, 6.6–26.4). There were five suicides among the deaths, the SMR for suicide being 10.7 (95% CI, 4.5–25.8).

**Table 5:** Causes of death during a 20-year follow-up of opioid addicts

**Cause of death**	**Females**	**SMR (95% CI)**	**Males**	**SMR (95% CI)**	**Total**	**SMR (95% CI)**
**Cardiovascular disease**	0	-	2	7.1 (1.8–28.4)	2	5.4 (1.3–21.5)
**Cancer**	2	5.1 (1.3–20.5)	1	3.3 (0.5–23.6)	3	4.3 (1.4–13.5)
**Other diseases**	15	63.8 (38.5–105.9)	35	66.7 (47.9–92.9)	50	65.8 (49.9–86.9)
**Accidents**	2	20.8 (5.2–83.2)	6	11.8 (5.3–26.2)	8	13.2 (6.6–26.4)
**Suicide**	3	25.9(8.3–80.2)	2	5.7 (1.4–22.9)	5	10.7 (4.5–25.8)
**Other violent deaths**	1	43.2 (6.1–306.7)	1	21.4 (3.0–151.8)	2	28.6(7.16–114.4)

**Total**	**23**	**24.2 (16.1–36.4)**	**47**	**23.4 (17.6–31.1)**	**70**	**23.6 (18.7–29.9)**

HIV/AIDS caused one death among the patients in this study (Table 4), and three deaths that occurred in 1990, 1992 and 1994 were classified as immune system disorders. Additional causes of death were bronchopneumonia, Hodgkin's disease and unspecified infectious or parasitic disease.

For the 37 deaths classified as drug dependence, an additional cause of death was mainly category 965, poisoning by analgesics, antipyretics and antirheumatics (ICD-9) accounting for 28 of the cases. For the rest of the group, acute or subacute endocarditis, unspecified bronchopneumonia, accidental poisoning by alcohol, accidental poisoning by barbiturates, and poisoning by other unspecified drugs or medicinal substances were listed.

In ICD-10, the drug dependence category per se does not exist. However, one death was classified as F11.0, psychiatric diseases and behavioural diseases caused by opiate dependence, and Hepatitis C and HIV were stated as additional causes of death. One death was classified as F19.0, psychiatric diseases and behavioural diseases caused by multiple drug dependence, with no additional cause of death listed. One death was classified as F12.0, mental and behavioural disorders due to use of cannabinoids, whereas chronic viral hepatitis B was an additional cause of death.

The numbers of deaths in each five-year period were too small to obtain cause-specific SMRs. However, causes of death were distributed evenly throughout the study period.

## Discussion

This prospective study followed all hospital-treated opioid addicts from the same large city, up to as long as 20 years. All patients were traced during this period, thus minimizing selection bias. Causes of death were obtained for all patients, enabling cause-specific mortality ratios to be determined so that they could be compared with those of the general population.

This study's main finding was the high mortality rate of 37.8%. One-third of females and almost half of males died. This high mortality rate was observed in a young patient group, for which the median age during 1980 and 1981 was 24 years. When corrected for the expected number versus the observed number of deaths, SMRs showed no statistically significant gender differences. Although in absolute numbers males had a higher mortality ratio than females, the expected number of deaths among males was higher as well. Therefore, the SMRs for males and females were almost identical, there being a 23-fold increase in mortality. In this study, the effect of opioid addiction seemed to overrule the effects of age and gender on mortality.

The SMR of 23.6, found for this cohort, is similar to SMRs obtained in other long-term European studies [1], although there were differences in the inclusion criteria for these studies. SMRs were 15 times higher for male drug users in Rome compared with the general population [2], 22 times higher for drug injectors in Glasgow [3], and 28.5 times higher for heroin addicts in Catalonia, Spain [4].

The lack of gender differences has also been observed in other studies, such as those conducted with homeless people who had a drug addiction [23]. However, other studies have found higher SMRs for either males [8,15] or females [2,13]. This diversity of findings may be due to different inclusion criteria. Gender differences have been observed when notified addicts or drug users, who were recruited from drug treatment centres, were included whereas the present study included hospital-treated opioid addicts. Therefore, this subject sample may have been more prone to accidental poisoning, such incidents being less correlated to gender perhaps than to behavioural traits, such as the pattern of drug use. Irrespective of toxic compound, the SMR for hospital-treated opioid addicts was much higher than that for hospital-treated self-poisonings. In a 20-year follow-up study of all self-poisonings treated in Oslo hospitals in 1980, the SMR was 4.6 (95% CI, 4.1–5.1), compared with 23.6 in the present cohort [24]. Opioid addicts are therefore at special risk. There were a higher number of deaths in the first five-year period of the study, and a decrease in SMR from 32.4 in the first five-year period to 13.4 in the last five-year period. It is suggested that those who take the highest risks will probably die early, leading to a decrease in mortality for the cohort as a whole. It has been shown that the number of active drug addicts declines mainly from death, rather than from long-term abstinence [25]. The decrease in SMR may also be due partly to increased mortality in the general population over time, leading to a relative reduction in the ratio.

There was an increased mortality for all causes of death among opioid addicts when compared with the general population, including both natural and unnatural causes of death. This was consistent with what is known about substance abusers in general [13]. The causes of death were mainly drug-related, as has been observed in other studies [3,15]. Five suicides occurred in the cohort. The increased risk of suicide among opioid addicts accords with other studies [9,10]. There has been controversy about the possible existence of a substantial number of hidden suicides among accidental poisonings. So far, the results support the hypothesis that most deliberate poisonings are accidental [26,27]. In the present study, the causes of death were obtained from Statistics Norway, and our group reported on the validity of these data in 1985 [28]. Since then, autopsy rates have been declining in Norway, and the reliability of death certificates has been questioned [29]. Therefore, the suicide numbers may well be too low. Mortality in the study population was compared with mortality rates from the whole country. Although the expected lifespan is lower in Oslo than the average for the country as a whole, it is less than one year below the average. Although SMR values found in the current study may be somewhat high, this will not change the study's major findings.

Only five deaths were registered as specific natural causes of death, three being cancer and two being cardiovascular diseases. The SMRs increased significantly, possibly due to confounding factors such as lower socio-economic status or tobacco use. Nevertheless, opioid addicts represent a group that is at high risk for excess mortality, not only from drug-related deaths, but also because of their increased risk of death from cancer and cardiovascular diseases. The increased risk of death, even from natural causes, is consistent with the increased mortality associated with other mental disorders [13].

Only one death could be classified as being caused by AIDS, although three deaths were stated as "disorders involving the immune mechanism". In other studies, AIDS has accounted for a majority of the deaths among opioid addicts [4]. A study of HIV-positive opioid addicts in Oslo found that drug overdose was a major cause of death, thus overriding the effect of AIDS on mortality [30].

The major cause of death was drug dependence, as registered in the other diseases category. In order to compare this cohort with the general population, we used the same categories as Statistics Norway. Although the SMR for this single cause of death was not calculated, this value would have provided minimal additional information. If drug dependence is a chronic disease, a symptom of which is opioid addiction, one would expect that only those suffering from the disease would die from it; that is, there would be a low number of deaths in the background population. Even when all other diseases were considered in the general population, death from drug dependence outnumbers the total number of deaths from all other diseases. As the classification of drug-related deaths is problematic in the ICD system, the term "drug-related deaths", used in mortality statistics, is currently being developed by the European Monitoring Centre for Drugs and Drug Addiction.

The category, drug dependence, is not equivalent literally to any category in ICD-10. Chapter X4 can be used for accidental poisonings, whereas F10 to F19 cover psychiatric and behavioural disorders caused by drug dependence. The incongruence of these classification systems makes it difficult to know if the categories cover the same spectrum of patients.

In this study, mortality did not change from referral to voluntarily detoxification. Our hypothesis was that those treated solely for self-poisonings would have a poorer prognosis, since such people were not sent to drug addiction units on a regular basis. Sorensen et al. found a significant decrease in mortality for those who achieved abstinence [7], but in the present study, it was not known whether patients completed or discontinued the detoxification programme. Those who joined the programme voluntarily may have also been in a poorer physical condition, since this was one of the criteria for admittance. Some of the patients admitted for detoxification were transferred to other units specialising in the treatment of addicts, whereas others were admitted for a few days of detoxification in the medical department because of their extremely poor medical and physical state. Generally, these patients were not motivated to partake in treatment required to achieve abstinence, and were among those with the most serious drug addiction problems. In addition, we did not have information about the completion rate for those transferred for further treatment.

Unfortunately, in retrospect, we were unable to trace the status of each patient at discharge, but could only do so for the group as a whole. However, the high level of mortality in both subgroups supports the hypothesis that referral to a detoxification programme alone is not sufficiently effective to prevent the excess mortality for this group. Patients probably need a longer and more closely supervised follow-up, both to improve their physical health and to achieve abstinence.

Since the relatively small numbers of deaths in each category did not make it meaningful to obtain cause-specific SMRs for each time period, it was not possible to investigate how cause-specific mortality ratios change over time. Subgroups might have been used in the statistical analyses to evaluate the effect of repeated treatments on mortality. However, the resulting groups would be too small to reveal any statistically significant differences. It might be worthwhile investigating in larger studies whether there could still be a difference between patients seeking repeated treatments and other patients.

The fact that mortality in this group was quite high when compared with the general population is a great challenge to our society. It is worth emphasizing that not all drug addicts die early. More research is needed to discover what makes those patients who survive different from those who die. This information would be helpful both for choosing therapy and for identifying those at special risk.

## Conclusion

The mortality rate was much higher among opioid addicts than in the general population, and most deaths were related to drug dependence. The risk of death was highest in the first five-year period, but the risk of death remained high throughout the whole 20-year follow-up period. There were no differences between those admitted for voluntary detoxification and those admitted solely for self-poisonings. Opioid addiction also seemed to override the effects of age and gender on mortality.

## Competing interests

The author(s) declare that they have no competing interests.

## Authors' contributions

MAB helped obtain information on each patient's status and their cause of death, participated in the design of the study, and drafted the manuscript. ASB helped obtain information on each patient's status and their cause of death, and helped draft the manuscript. AO helped obtain information on each patient's status and their cause of death, and helped draft the manuscript. TH performed the statistical analyses. DJ obtained the cohort of hospitalized opioid addicts admitted for self-poisoning and participated in the design of the study. MR obtained the cohort of patients admitted for voluntarily detoxification. OE conceived of the study, participated in its design and coordination, and helped draft the manuscript. All authors read and approved the final version of the manuscript.

## Pre-publication history

The pre-publication history for this paper can be accessed here:


